# Single‐nucleotide polymorphism at alcohol dehydrogenase 1B: A susceptible gene marker in oro‐/hypopharyngeal cancers from genome‐wide association study

**DOI:** 10.1002/cam4.6506

**Published:** 2023-09-14

**Authors:** Hui‐Tzu Chien, Chia‐Lung Tsai, Chi‐Kuan Young, Yun‐Shien Lee, Chun‐Ta Liao, Chih‐Ching Yeh, Angel Chao, Kai‐Lun Cho, Ching‐Han Chen, Shiang‐Fu Huang

**Affiliations:** ^1^ Department of Nutrition and Health Sciences Chang Gung University of Science and Technology Taoyuan Taiwan; ^2^ Geriatric and Long‐Term Care Research Center Chang Gung University of Science and Technology Taoyuan Taiwan; ^3^ Genomic Medicine Research Core Laboratory Chang Gung Memorial Hospital, Linkou Branch Taoyuan Taiwan; ^4^ Department of Otolaryngology, Head and Neck Surgery Chang Gung Memorial Hospital, Keelung Branch Keelung Taiwan; ^5^ Medical College, Chang Gung Memorial Hospital Taoyuan Taiwan; ^6^ Department of Biotechnology Ming Chuan University Taoyuan Taiwan; ^7^ Department of Otolaryngology, Head and Neck Surgery Chang Gung Memorial Hospital Linkou Taiwan; ^8^ Master Program in Applied Molecular Epidemiology, College of Public Health Taipei Medical University Taipei Taiwan; ^9^ School of Public Health, College of Public Health Taipei Medical University Taipei Taiwan; ^10^ Cancer Center, Wan Fang Hospital Taipei Medical University Taipei Taiwan; ^11^ Department of Obstetrics and Gynecology Chang Gung Memorial Hospital and Chang Gung University Taoyuan Taiwan; ^12^ School of Medicine, Chang Gung Medical College Chang Gung University Taoyuan Taiwan; ^13^ Graduate Institute of Clinical Medical Sciences Chang Gung University Taoyuan Taiwan

**Keywords:** alcohol dehydrogenase 1B, aldehyde dehydrogenase 2, genome‐wide association study, head and neck cancer, oropharyngeal cancer

## Abstract

**Introduction:**

In the era of precision preventive medicine, susceptible genetic markers for oro‐/hypopharyngeal squamous cell carcinoma (OPSCC) have been investigated for genome‐wide associations.

**Materials and Methods:**

A case–control study including 659 male head and neck squamous cell carcinoma (HNSCC) patients, including 331 oropharyngeal cancer, treated between March 1996 and December 2016 and 2400 normal controls was performed. A single‐nucleotide polymorphism (SNP) array was used to determine genetic loci that increase susceptibility to OPSCC.

**Results:**

We analyzed the allele frequencies of 664,994 autosomal SNPs in 659 HNSCC cases; 7 SNPs scattered in loci of chromosomes 5, 7, 9, 11, and 19 were significant in genome‐wide association analysis (*Pc* < 1.0669 × 10^−7^). In OPSCCs (*n* = 331), two clustered regions in chromosomes 4 and 6 were significantly different from the controls. We successfully identified a missense alteration of the SNP region in alcohol dehydrogenase 1B (*ADH1B*) (https://genome.ucsc.edu; hg38); the top correlated locus was rs1229984 (*p* = 1 × 10^−11^). Adjusted for environmental exposure, including smoking, alcohol, and areca quid, a region in chromosome 12, related to alcohol metabolism, was found to independently increase the susceptibility to OPSCC. The *ADH1B* rs1229984 AA genotype had better overall survival compared to the AG and GG genotypes (*p* = 0.042) in OPSCC. The GG genotype in rs1229984 was significantly associated with a younger age of onset than other genotypes (*p* = 0.001 and <0.001, respectively) in OPSCC patients who consumed alcohol.

**Conclusion:**

*ADH1B* was an important genetic locus that significantly correlated with the development of OPSCCs and patient survival.

## Graphical abstract

This is a large genome wide association study for head and neck squamous cell carcinoma (HNSCC) in areca‐quid endemic region. Germline genetic variations in oro‐/hypopharyngeal cancer different from other HNSCCs. Alcohol dehydrogenase 1B (ADH1B) single‐nucleotide polymorphism (SNP) important in the susceptibility of oro‐ and hypopharyngeal cancer.

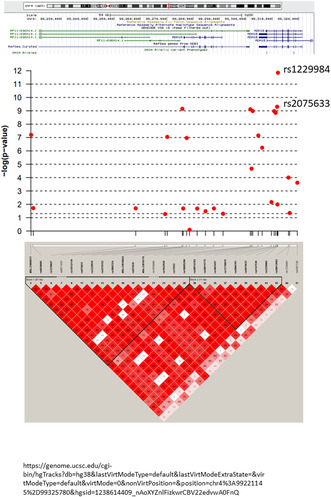

## 1 INTRODUCTION

Head and neck squamous cell carcinomas (HNSCCs) ranked the sixth highest incidence of all cancers worldwide and the majority were oral cavity cancers. They are the fourth most common malignancies among Taiwanese males^1^ and their incidence is increasing. Oro‐/hypopharyngeal squamous cell carcinoma (OPSCC) is a subgroup of HNSCCs. In Taiwan, the occurrence of OPSCC is approximately 941 new cases (11.47% in HNSCC) and 400 deaths in 2019 (https://www.hpa.gov.tw/Pages/Detail.aspx?nodeid=269&pid=14913). The treatments such as surgery, irradiation, and chemotherapy of OPSCC usually accompany with swallowing and speech sequalae. Early detection of OPSCC significantly improves the survival rate and functional outcome.

The tumorigenesis of HNSCCs is closely associated with tobacco, areca quid (AQ), alcohol consumption.^2^, ^3^ Human papilloma virus (HPV) infection recently was identified to play important roles in the carcinogenesis of oropharyngeal cancer. More importantly, HPV infection in oropharyngeal cancer patients renders a better prognosis after chemoradiation therapy.^4^ In addition to the environmental exposures, individuals' susceptibility was investigated to delineate the complex host–environment interactions. In our previous study, familial aggregation was observed for HNSCCs including OPSCCs in a population‐based analyses,^5^ suggesting the role of genetics in the process of tumor development. In oropharyngeal cancers, genome‐wide association analyses identify several loci, including rs1229984 in *ADH1B*, rs3828805 in HLA‐DQB1, rs4318431 nearby gene *GALNT14*, rs13211972 in *MUC21*, and rs34518860 in HLA‐DQA1, are correlated with patients' susceptibility and prognosis.^6^, ^7^ In the literature, studies on genetic susceptibility in AQ endemic regions focused on oral cavity cancer. The OPSCC genetic studies in AQ use area were limited and the case numbers in these studies were 85 patients in Japan and 103 patients in Taiwan.^8^, ^9^ The knowledge of susceptible genes in OPSCC could be helpful clinically. Early detection of oropharyngeal and esophageal lesions is challenging. Utilizing genetics in picking out susceptible OPSCC individuals could improve the screening and early detection in a cost‐effective manner.^10^

To identify high‐risk genetic loci for OPSCC in Taiwan, we retrospectively investigated 697 HNSCC patients, including 331 OPSCCs, 345 oral cavity and other subsites. A questionnaire was used to collect detailed information about environmental exposures. Single‐nucleotide polymorphism (SNP) arrays and environmental exposure adjustments were utilized to find high‐risk loci for OPSCCs.

## 2 MATERIALS AND METHODS

### 2.1 Study population

We recruited 697 male HNSCC patients including 331 OPSCC treated at Chang Gung Memorial Hospital, Lin‐Kou, Taiwan in the period between 1996 and 2016. All patients were a histologically confirmed of primary squamous cell carcinoma.

As per the 2010 guidelines from the American Joint Committee on Cancer, oral cavity cancer was characterized as cancer occurring in the lip, buccal mucosa, alveolus, retromolar area, tongue, floor of the mouth, or hard palate.^11^ Oropharyngeal cancer encompassed the subsites of the soft palate, oropharyngeal walls, or tonsils.^11^ Similarly, hypopharyngeal cancer was defined as cancer located in the lower portion of the pharynx and included the lateral pharyngeal walls, pyriform sinus, and posterior cricoid region.^11^

All patients were followed up regularly for >2 years. A questionnaire was employed to gather details pertaining to demographic information, familial background, and behaviors encompassing cigarette smoking, alcohol consumption, and areca nut (AQ) usage. Individuals were categorized as smokers if they had smoked more than 100 cigarettes throughout their lifetime. Those who imbibed alcohol at least once a month were classified as alcohol drinkers. Consistent AQ chewers were identified as those who had consumed over 100 nuts in their lifetime.

### 2.2 Participant selection

This study was approved by the Institutional Review Board of Chang Gung Medical Foundation (201800439B0). We recruited 2400 ethnically and geographically matched healthy controls (TMU‐201805076) from a biobank as a nationwide population study.^12^ Informed consent was obtained from all participants. The controls were recruited from Taiwan. Most (98%) of the population is Han Chinese and few were Hakka Chinese. Furthermore, 100 healthy controls were randomly selected for genotype validation.^13^

### 2.3 Specimen collection and DNA extraction

In all participants, a 10 mL sample of venous blood was collected into a vacuum tube containing an anticoagulant (Vacutainer; BD). The buffy coat, obtained from this sample, was isolated and stored at a temperature of −80°C. High‐molecular weight DNA was then extracted from the buffy coat cells through the employment of the phenol–chloroform method and subsequently stored at −80°C.^13^

### 2.4 Genotyping and quality control

A genome‐wide association study (GWAS) of samples containing 703,949 SNPs obtained from 697 HNSCC patients and 2400 controls was performed using the Axiom Genome‐Wide TWB Array Plate (Affymetrix GeneTitan; Thermo Fisher Scientific).^14^ To evaluate DNA quantity and purity, a NanoDrop ND‐1000 spectrophotometer (NanoDrop Technologies LLC) was used; an absorbance ratio of 260/280 and a purity index >1.8 were considered optimal. The volume for array analysis was 50 μL at a concentration of 15 ng/μL for all samples. The GWAS dataset underwent analysis using the PLINK software (v1.90b5). Logistic regression was executed, incorporating sex and ancestry‐specific principal components (referred to as PC1–PC10) as covariates.^15^, ^16^ For genomic coordinates, the National Center for Biotechnology Information Human Genome Build 37 (GRCh37) was utilized. Out of the 703,949 genotyped SNPs, 664,994 situated on autosomal chromosomes underwent quality control. SNPs with a call rate exceeding 0.95 were retained, whereas 47,488 variants were excluded due to missing genotype information. A total of 143,017 variants were discarded due to their low minor allele frequency (<0.01), and 5849 were eliminated for deviating from Hardy–Weinberg equilibrium. To visualize potential deviations from expected distributions, a quantile‐quantile plot was generated using the Bioconductor package GWASTool in R language (The R Foundation).^17^ The genomic inflation factor was computed through PLINK. Linkage disequilibrium analyses for the SNPs rs1229984 and rs671 were conducted using the LDproxy module available in the online software LDLink (https://analysistools.nci.nih.gov/LDlink). As the genotyped SNPs on the TWB Array totaled 703,949 and had been preselected and filtered based on their frequencies in Asian populations, no further genotype imputation was done in our study.

### 2.5 SNP array genotyping and quality control

Quality control procedures were executed both at the individual and marker levels. Initial assessments encompassed individual‐specific aspects, such as sample quality, kinship, and population stratification. Dish‐sample quality control (DQC) was employed to oversee non‐polymorphic sites, gauging signal and background channels. Individuals falling below satisfactory DQC thresholds and maintaining call rates of less than 97% were excluded.

Additionally, a plate pass rate was introduced, involving the selection of samples with acceptable DQC values and a call rate of 97% or higher, divided by the total number of samples on the respective plate. Inclusion criteria for analysis encompassed samples boasting a call pass rate surpassing 95%, with an average call rate of sample passage exceeding 99%. To address inbreeding, coefficients were evaluated, and samples displaying significant kinship connections were removed. The genome‐wide identity was investigated through multidimensional scaling analysis, facilitating the identification and removal of outlier clusters.

In relation to markers, markers failing to meet specific criteria were omitted. These criteria included a missing rate below 2%, a minor allele frequency exceeding 1%, and adherence to Hardy–Weinberg equilibrium (*p* > 0.001). The replication sample underwent an identical set of quality control procedures to ensure consistency and reliability.

### 2.6 *ADH1B* and *ALDH2* genotyping

In the replication study, direct sequencing of associated SNPs on the initial GWAS was performed using an independent sample set of 100 HNSCC cases and controls each. Genotyping for *ALDH2*, rs671, and rs1229984, was done by direct sequencing. The wild‐type allele ALDH2*1 and variant allele*2 were defined as Glu504 and Lys504 (rs671), respectively. Polymerase chain reaction (PCR) amplification of the SNP rs671 near *ALDH2* was performed using the forward primer 5′‐TCCTATTGCATTGGGCATATT‐3′ and reverse primer 5′‐TCCATTTACGCCTCAACTCA‐3′. The forward and reverse primers for rs1229984 were 5′‐TCACCCCTTCTCCAACACTC‐3′ and 5′‐ATTCTGTAGATGGTGGCTGTAG‐3′. The annealing temperature for both rs671 and rs1229984 was 58°C. The conditions for PCR reactions were as previously described.^18^

### 2.7 Statistical analysis

The distributions of age, tumor subsites, cigarette smoking, AQ chewing, and alcohol consumption among HNSCC patients were calculated. Chi‐squared test and *t*‐test were used for the analyses of categorical and continuous variables, respectively. *p* < 0.05 were considered significant. A multivariate analysis was performed using Cox regression. The curves for the age at disease onset were compared between genotypes using the Kolmogorov–Smirnov test.^19^ Overall survival (OS) and disease‐free survival (DFS) were assessed using the Kaplan–Meier method and differences were estimated using the logrank test. All analyses were performed using SPSS Statistics version 18 (IBM Corp.) and R language (Version 4.2.2, The R Foundation).^20^

## 3 RESULTS

A GWAS of 697 HNSCC patients and 2400 controls was initially performed (Table 1); 703,949 variants were analyzed using the TW2.0 SNP chip. After quality control and removal of variants with minor allele frequencies <1%, 468,640 variants and 3085 samples (including 697 cases and 2400 controls) were included. SNPs on sex chromosomes were excluded from the GWAS.

**TABLE 1:** Tumor subsite distributions in the study cohort (*n* = 698).

	All patients (*n* = 697)^a^		Normal population (*n* = 2400)
Age (mean ± SD)	52.43 (±10.75)		50.02 (±10.58)
Range	25–88		30–69
Oral cavity	345	49.4%	
Oropharynx	147	21.1%	
Hypopharynx	184	26.4%	
Larynx	12	1.7%	
Nasal cavity	3	0.4%	
Unknow primary	2	0.3%	
Esophagus	4	0.6%	
Oropharynx (*n* = 331)			
Tonsil	57	17.2%	
Tongue base	36	10.9%	
Soft palate	42	12.7%	
Lateral pharyngeal wall	23	6.9%	
Pyriform sinus	145	43.8%	
Posterior pharyngeal wall	28	8.5%	
Oral cavity cancer (*n* = 345)			
Tongue	101	29.3%	
Mouth floor	13	3.8%	
Lip	13	3.8%	
Buccal mucosa	115	33.3%	
Alveolus	26	7.5%	
Hard palate	10	2.9%	
Retromolar trigone	15	4.3%	
Multiple primary cancers	52	15.1%	

Abbreviation: SD, standard deviation.

^a^ One skin cancer patient was removed during analysis.

**FIGURE 1:**
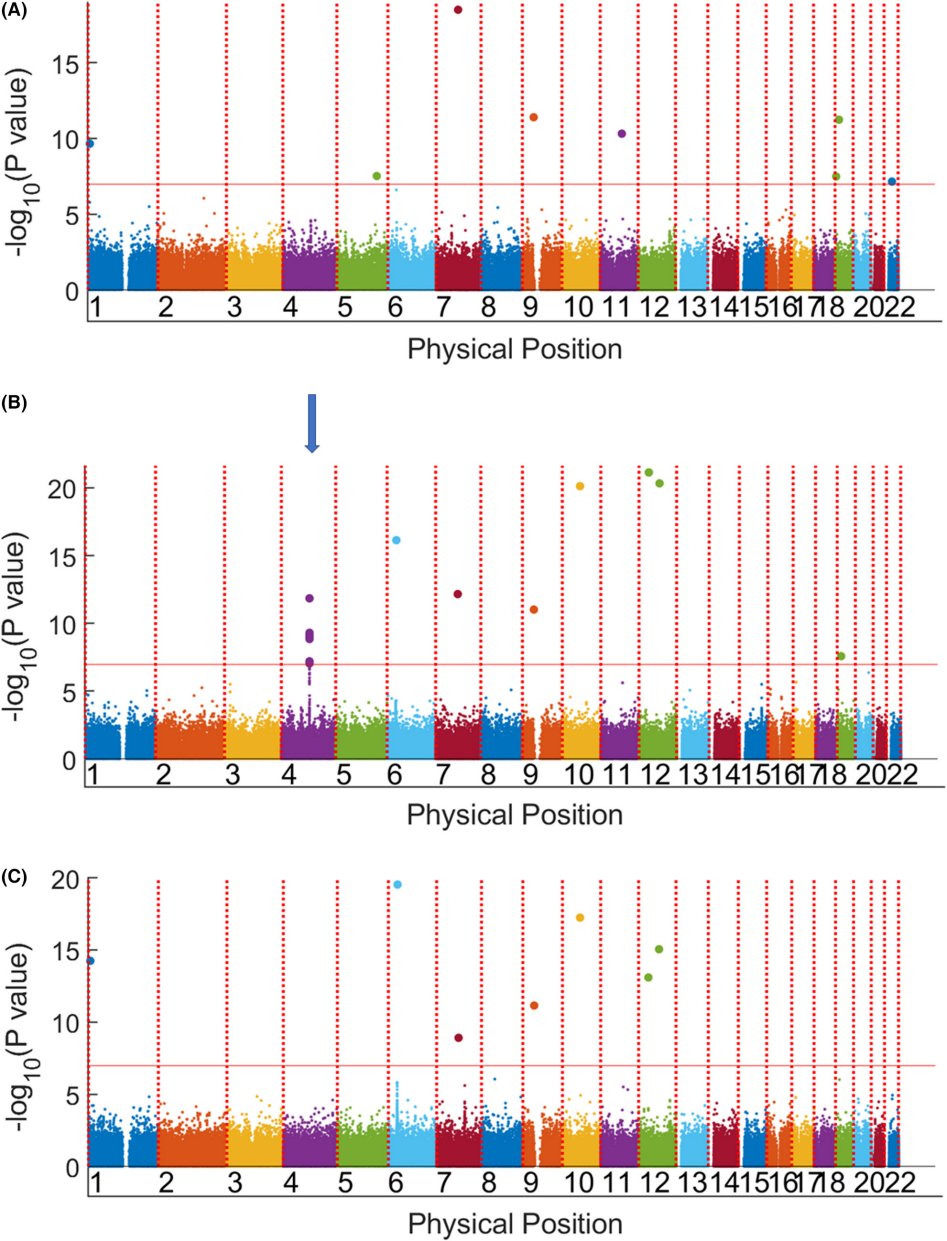
Manhattan plot of the genome‐wide association results. The *y*‐axis corresponds to −log10 *p*‐values and the *x*‐axis corresponds to the genomic positions. The horizontal red line (*p* = 5 × 10^−8^) denotes the false discovery rate. (A) Head and neck squamous cell carcinomas. (B) Oro‐/hypopharyngeal squamous cell carcinomas (OPSCCs). (C) Oral cavity squamous cell carcinoma. A clustered region of single‐nucleotide polymorphisms in chromosome 4 (blue arrow) were found in OPSCCs (B).

The principal component analysis map showed no differences in the distribution of ancestry between HNSCC patients and controls (Figure S1). A quantile‐quantile plot used for quality control demonstrated successful population matching (Figure S2). The genomic inflation factor (λGC)^21^ was 1.061, suggesting an acceptable population structure in the GWAS. Figure 1 shows the Manhattan plot according to tumor subsites. When comparing allele frequencies of the 664,994 autosomal SNPs in 331 OPSCC cases and 2400 controls, 13 SNPs reached the threshold of genome‐wide significance (*Pc* < 1.0669 × 10^7^) (Table S1).

**FIGURE 2:**
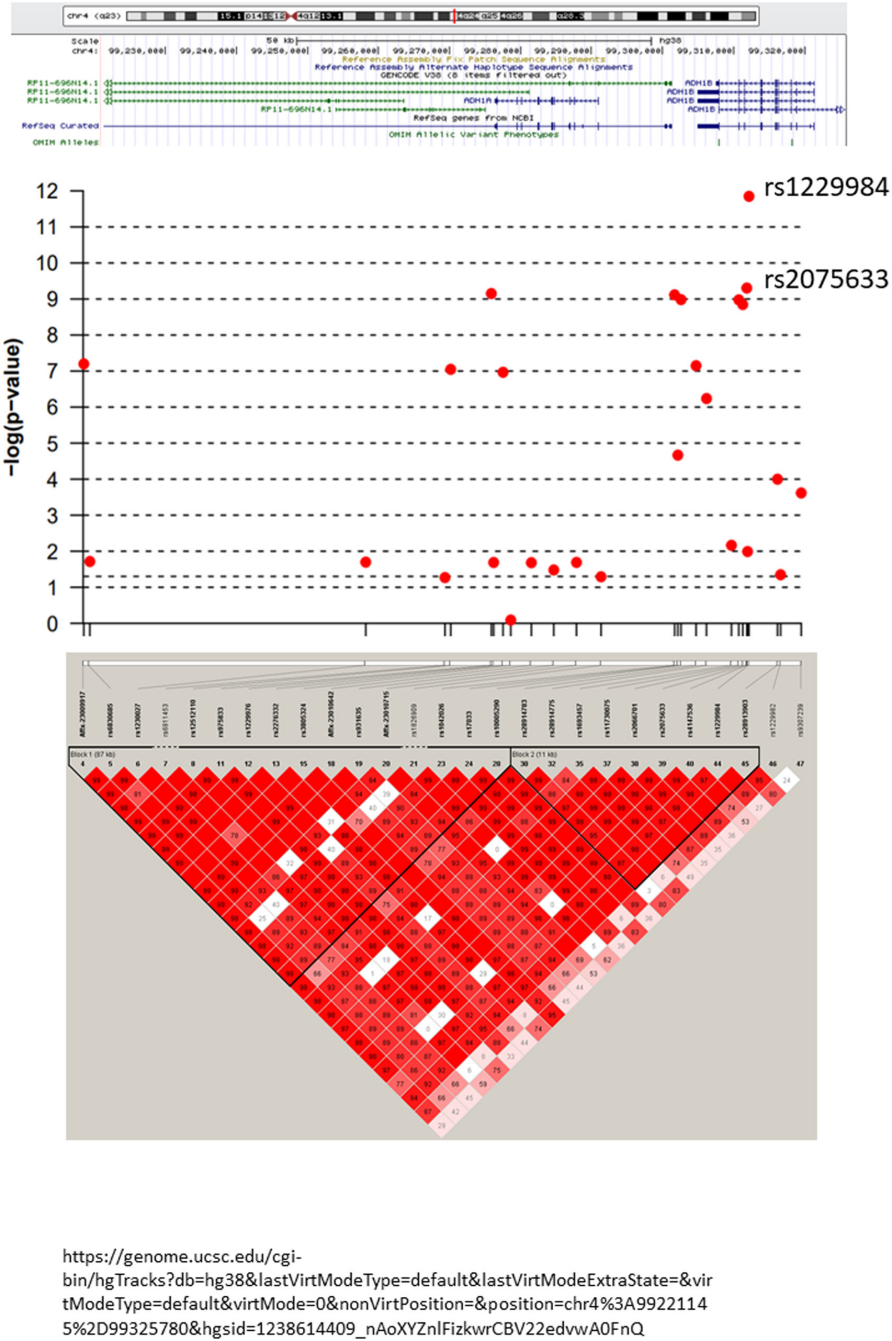
Linkage disequilibrium diagram of chromosome 4. We successfully identified a missense alteration of the single‐nucleotide polymorphism region in alcohol dehydrogenase 1B (https://genome.ucsc.edu; hg38); the top correlated locus was rs1229984 (*p* = 1 × 10^−11^).

**FIGURE 3:**
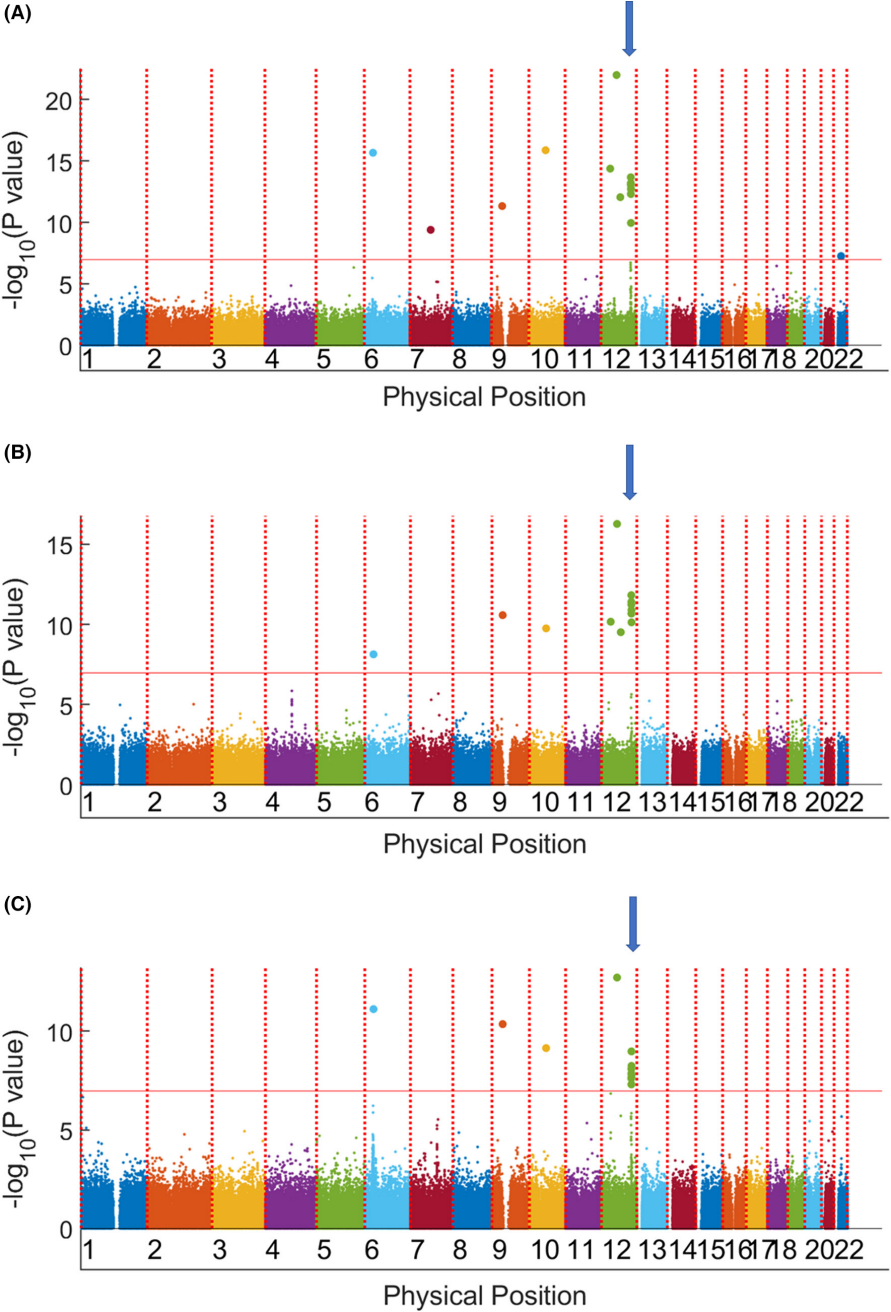
Manhattan plot of the genome‐wide association results after adjustment of environmental exposures of alcohol, smoking, and areca quid. Chromosome 12 was significantly related with all head and neck cancers (arrow). (A) Head and neck squamous cell carcinomas. (B) Oro‐/hypopharyngeal squamous cell carcinomas. (C) Oral cavity cancers.

**FIGURE 4:**
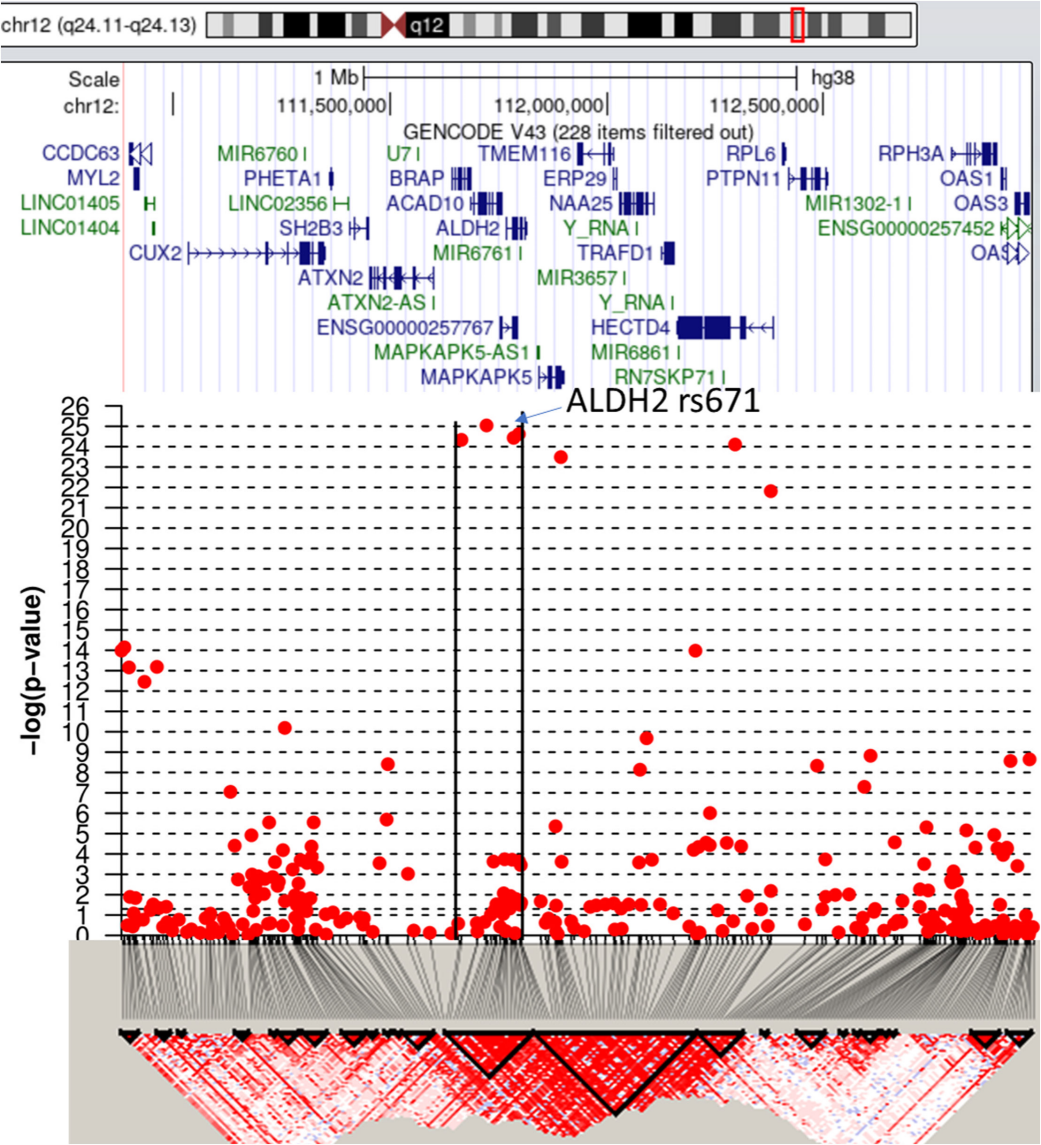
Linkage disequilibrium diagram of chromosome 12. We successfully identified a missense alteration of the single‐nucleotide polymorphism region in aldehyde dehydrogenase 2 (*ALDH2*) (https://genome.ucsc.edu; hg38); the top correlated locus was rs671 (*p* = 1 × 10^−25^).

A cut‐off *p*‐value of 1 × 10^−7^ was determined by false discovery rate. In all HNSCC patients (*n* = 697), only scattered loci in chromosomes 7, 9, 11, and 19 were identified as significant (Table 1, Figure 1). Similar scattered loci were found in the oral cavity cancer subgroup (*n* = 293) compared to the controls. In the OPSCC subgroup (*n* = 331), two regions located in chromosomes 4 and 6 were different compared to the controls. We successfully identified a missense alteration in the SNP region of *ADH1B* (https://genome.ucsc.edu; hg38); the top correlated locus was rs1229984 (Figure 2; *p* = 1 × 10^−11^). Adjustment for environmental exposure, including cigarettes, alcohol, and AQ, led to the identification of a region of chromosome 12 for susceptibility to OPSCC (Figure 3). We further identified a missense alteration in the SNP region of *ALDH2* (https://genome.ucsc.edu; hg38); the top correlated locus was rs671 (Figure 4, *p* = 1 × 10^−25^). The sequence results of rs671 and rs1229984 in 100 samples matched completely with the array genotyping.

To investigate the effect of rs671 and rs1229984 in HNSCC carcinogenesis, univariate analysis with different models were analyzed (Table 2). We can see the OPSCC carries the highest hazard ratio (HR) with rs671 (1.920, 95% confidence interval [95% CI]: 1.512–2.439) and rs1229984 (1.714, 95% CI: 1.356–2.167) in dominant model compared with HNSCC and OSCC. Multiple logistic regression analyses were also performed to adjust for environmental factors, including cigarette, alcohol, and AQ use (Table 3).

**TABLE 2:** Nonsynonymous SNPs in *ADH1B* (rs671) and *ALDH2* (rs1229984) and head and neck cancer, oro‐/hypopharyngeal cancer and oral squamous cell carcinoma in univariate analysis.

	Normal (*n* = 2388)	HNSCC (*n* = 697)	HNSCC (HR [95% CI])	OPSCC (*n* = 331)	OPSCC (HR [95% CI])	OSCC (*n* = 293)	OSCC (HR [95% CI])
rs671							
GG	1223	263	**<0.001**	117	**<0.001**	117	**0.001**
GA	984	407		211		151	
AA	181	27		3		25	
Dominant model	1165	434	**<0.001**	214	**<0.001**	176	**<0.001**
(AA+GA vs. GG)	1223	263	1.732 (1.457–2.059)	117	1.920 (1.512–2.439)	117	1.579 (1.233–2.022)
Recessive model	181	27	**<0.001**	3	**<0.001**	25	0.563
(AA vs. GA + GG)	2207	670	0.491 (0.325–0.743)	328	0.111 (0.035–0.351)	268	1.137 (0.735–1.761)
Overdominant model	984	407	**<0.001**	211	**<0.001**	151	**<0001**
(GA vs. GG + AA)	1404	290	2.002 (1.687–2.376)	120	2.509 (1.977–3.184)	142	1.517 (1.190–1.935)
rs1229984							
AA	1269	346	**<0.001**	132	**<0.001**	176	0.077
AG	943	251		128		97	
GG	173	100		71		20	
Dominant model	1116	351	0.101	199	**<0.001**	117	**0.026**
(GA + GG vs. AA)	1269	346	1.153 (0.974–1.366)	132	1.714 (1.356–2.167)	176	0.756 (0.590–0.968)
Recessive model	173	100	**<0.001**	71	**<0.001**	20	0.789
(GG vs. GA + AA)	2212	597	2.141 (1.648–2.784)	260	3.492 (2.575–4.735)	273	0.937 (0.580–1.513)
Overdominant model	943	251	0.093	128	0.762	97	**0.033**
(AG vs. AA+GG)	1442	446	0.861 (0.722–1.025)	203	0.964 (0.762–1.221)	196	0.757 (0.585–0.978)

Abbreviations: HNSCC, head and neck squamous cell carcinoma; HR, hazard ratio; OPSCC, oro‐/hypo‐ pharyngeal squamous cell carcinoma; OSCC, oral squamous cell carcinoma.

The bold values stand for *p* value < 0.05.

**TABLE 3:** The hazard ratio of rs671 and rs1229984 in the tumorigenesis after logistic regression adjusting environmental exposure in different tumor locations.

	HNSqCC		Oro‐/hypopharynx cancer		Oral cavity cancer	
	HR (95% CI)	*p* value	HR (95% CI)	*p* value	HR (95% CI)	*p* value
rs671		**<0.001**		**<0.0001**		**<0.001**
G allele	1		1		1	
A allele	2.078 (1.694–2.549)		2.165 (1.666–2.814)		2.113 (1.635–2.730)	
rs1229984		0.062		**<0.0001**		0.245
T allele	1		1		1	
C allele	1.179 (0.992–1.402)		1.648 (1.335–2.034)		0.885 (0.719–1.088)	
Cigarette smoking		**<0.001**		**<0.0001**		0.051
No	1		1		1	
Yes	2.562 (1.819–3.608)		2.494 (1.585–3.924)		1.569 (0.998–2.466)	
Alcohol drinking		**<0.001**		**<0.0001**		**<0.001**
No	1		1		1	
Yes	9.278 (7.141–12.056)		13.123 (9.152–18.815)		6.424 (4.570–9.031)	
AQ chewing		**<0.001**		**<0.0001**		**<0.001**
No	1		1		1	
Yes	7.989 (6.174–10.337)		4.628 (3.347–6.399)		14.212 (9.579–21.085)	

Abbreviations: AQ, areca‐quid chewing; CI, confidence interval; HNSqCC, head and neck squamous cell carcinoma; HR, hazard ratio.

The bold values stand for *p* value < 0.05.

### 3.1 Clinical implications

Alcohol, cigarettes, and AQ are the three most common environmental causes of HNSCCs. To clarify the role of rs671 and rs1229984 in HNSCC tumorigenesis, we adjusted for these environmental factors using logistic regression (Table 3). We demonstrated that rs671 was independently related to both OPSCC and oral cavity cancers (odds ratio [OR]: 2.078–2.165), while rs1229984 was only related to OPSCCs (OR: 1.648, 95% CI: 1.335–2.034). AQ users had the highest risk (OR: 14.212, 95% CI: 9.579–21.085) of oral cavity cancer, while alcohol played a more important role in OPSCCs (OR: 13.123, 95% CI: 9.152–18.815). This suggested that alcohol consumption and genotypes of *ADH1B* and *ALDH2* may be used as predictors of susceptibility to OPSCCs.

### 3.2 Survival

**FIGURE 5:**
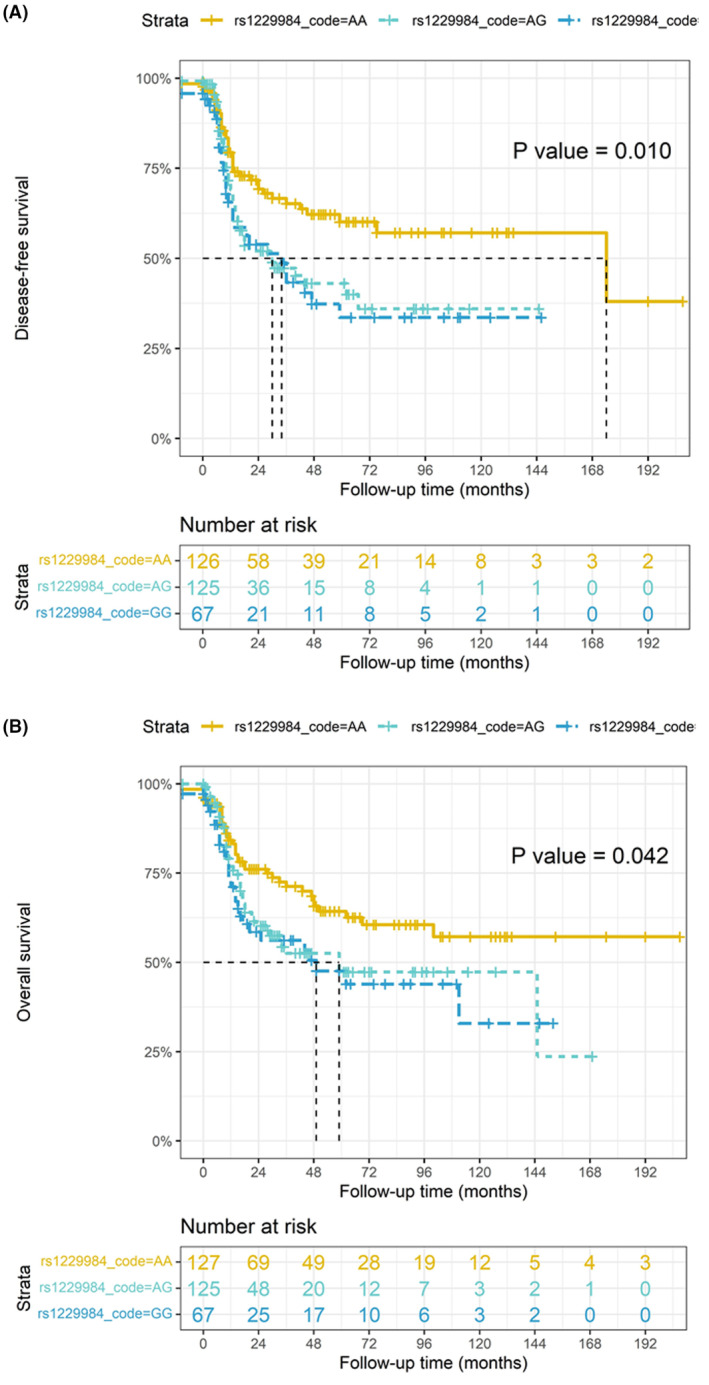
(A) In oro‐/hypopharyngeal squamous cell carcinomas, the alcohol dehydrogenase 1B rs1229984 GG genotype had better disease‐free survival (DFS) compared to the AA and AG genotypes (*p* = 0.010). (B) The GG genotype had better overall survival (OS) compared to the AA and AG genotypes (*p* = 0.042).

In OPSCC, *ADH1B* rs1229984 had significantly different DFS (Figure 5A, *p* = 0.010) and the GG genotype had better OS compared to the AA and AG genotypes (Figure 5B, *p* = 0.042). Meanwhile, *ALDH2* (rs671) genotypes had no significant effects on DFS or OS (*p* = 0.274 and 0.711, respectively). In all HNSCCs, *ALDH2* genotypes did not influence DFS (*p* = 0.325) and OS (*p* = 0.163). *ADH1B* had significantly different OS (*p* = 0.030), but did not influence DFS (*p* = 0.786). In oral cavity cancers, *ADH1B* and *ALDH2* had no effects on DFS (*p* = 0.053 and 0.607, respectively) and OS (*p* = 0.433 and 0.361, respectively). In summary, the *ADH1B* genotype significantly influenced survival in OPSCC patients.

The correlation between the two genotypes (rs1229984 and rs671) and age at onset of OPSCC was further analyzed. The average ages of onset for rs1229984 AA (*n* = 132), AG (*n* = 128), and GG (*n* = 71) were 54.67 (standard deviation [SD]: ±9.290), 55.96 (SD: ±10.717), and 49.59 years (SD: ±9.845), respectively. The GG genotype had a significantly lower age of onset for OPSCCs among alcohol users compared to other genotypes (*p* = 0.001 and <0.001, respectively). The average ages at onset for rs671 GG (*n* = 117), GA (*n* = 221), and AA (*n* = 3) were 56.61 (SD: ±11.077), 52.48 (SD: ±9.248), and 68.00 years (SD: ±16.523), respectively. The AA genotype was associated with a higher age at onset compared to the GA genotype (*p* = 0.008); however, there were few rs671 AA genotype cases.

## 4 DISCUSSION

In the literature, limited risk gene loci were reported in OPSCC. It is due to the lower incidence of OPSCCs compared with other HNSCCs. Previous studies reported five susceptible regions, including rs1229984, rs3828805, rs4318431, rs13211972, and rs34518860, for OPSCC in European and American.^6^, ^7^ In this study, we identified two susceptible loci, rs1229984 and rs671, for the OPSCC in Chinese Han population. Among these loci, rs1229984 can be one very important genetic locus in OPSCC both in Asians, Europeans, and Americans.

Both two enzymes, *ADH1B* (rs1229984) and *ALDH2* (rs671), involve in alcohol metabolic pathway. Initially, alcohol is catalyzed to acetaldehyde by ADH, and then converted to acetate by ALDH to decrease cytotoxic stress. Previous study demonstrates a causal effect of smoking and alcohol on oral cancer and OPSCC.^22^ In this OPSCC GWAS study, we identified the *ADH1B* (rs1229984) as well as *ALDH2* (rs671) increase the risks associated with OPSCC in Chinese Han population.^6^ Our results may provide genetic evidence for the association between alcohol consumption and OPSCC.

Previous studies have aimed to understand individual susceptibility to environmental exposure by investigating gene–environment interactions, particularly for genetic markers that increase the risk for HNSCCs. Genetic studies have focused on carcinogen metabolism and the capability for DNA repair. Various genes, including *XRCC1* ^23^ and *CYP1B1* (rs10012 and rs1056836),^24^ have been implicated in HNSCC pathogenesis. These genes are mainly involved in the maintenance of genetic integrity or carcinogen metabolism.

In a meta‐analysis of aerodigestive tract squamous cell carcinomas in patients of European ancestry based on a GWAS, *ADH1B* was found to play a significant role in oral and oropharyngeal cancer development. The T allele was significantly protective against oral and oropharyngeal cancers compared to the C allele (OR: 0.58, 95% CI: 0.50–0.67). In our previous study, a functional genetic polymorphism of the T allele in *ADH1B* was also associated with the occurrence of multiple upper aerodigestive tract primary tumors.^18^ No other studies have demonstrated an association of *ADH1B* and *ALDH2* with OPSCCs. From Figure 1, we can see the genetic effects of *ADH1B* and *ALDH2* were easily obscured in HNSCCs. When we stratified the population into different tumor subsites, the associations between *ADH1B*, *ALDH2*, and OPSCC became more and more evident.

Alcohol has a demonstrated association with esophageal cancers^25^; its effects are more important in upper digestive tract carcinogenesis. These effects are caused by alcohol metabolites and because ethanol acts as a solvent for carcinogens that cause upper aerodigestive tract cancers.^26^, ^27^ The effects of alcohol are more prominent among Asians because of alcohol flush reactions, related to the reduced activity of alcohol‐metabolizing enzymes. Surprisingly, the carcinogenic effects of alcohol‐metabolizing enzymes emerged as critical loci in HNSCCs and their influence was independent of exposure to cigarettes, alcohol, and AQ.

From our analysis, we found that the effects of *ADH1B* and *ALDH2* are not limited to cancer onset, but also contribute to cancer prognosis.^28^, ^29^ The prognostic value of *ADH1B* was seldom investigated in the literature. *ADH1B* (rs122984) was associated better OS in HNSCC (*p* = 0.030) and OPSCC (*p* = 0.042) patients, but not in oral cavity cancer patients (*p* = 0.433). The effect of *ADH1B* on HNSCC survival has rarely been reported before. Lee et al. found that pre‐diagnosis alcohol consumption was significantly related with worse OS of HNSCC patients. *ADH1B* and *ALDH2* modified the relationship between alcohol use and OS of HNSCC patients.^30^ Kagemoto et al.^28^ demonstrated that *ADH1B* and *ALDH2* were related to survival in esophageal cancer patients.^18^ Lee et al. speculated that the influence of alcohol, *ADH1B* and *ALDH2* on OS could be related with the advanced stage of HNSCC.^30^ We have previously demonstrated that *ADH1B* (*1 allele carriers) significantly increase the risk of developing multiple primary tumors in the upper digestive tract (OR, 2.093; 95% CI: 1.149–3.812).^18^ The influence of survival by *AHD1B* could come from the increased risks of multiple primary tumors in susceptible genetic carriers. The underlying mechanisms need to be investigated in a larger population and longer follow‐up period.

HPV infection was reported to play important roles in oropharyngeal cancers.^31^ The limitation of this study is lack of adjustment of HPV infection in OPSCCs. We used PCR‐based method to detect HPV infection in a small subset of OPSCCs in this study (*n* = 144).^32^ The rate of HPV infection OPSCCs was low (2.1%, data not shown). Samples in our study were recruited since 1996 and the infection rate in OPSCCs could be low in patients recruited two decades ago.^31^ From our study, it proves the genetics also play a role in the tumor formation in HNSCCs. Although OSCC and OPSCC located within the field of head and neck, we found that the susceptible gene loci in OPSCCs are different from OSCCs. Alcohol‐metabolizing genes are more important in the tumor formation in OPSCCs than in OSCCs. *ADH1B* and *ALDH2*, among all alcohol‐metabolizing genes, were found to play critical roles in the development of OPSCCs. In Asian population, susceptible genes loci could be utilized to increase the efficacy of screening in general population.

## 5 CONCLUSIONS

The present GWAS of OPSCC included one of the largest cohorts in the Asian population. In contrast to Western population, *ADH1B* and *ALDH2* were found to play critical roles in the development of OPSCCs. *ADH1B* had greater effects on OPSCCs than *ALDH2* in terms of age at onset and survival rates. From the perspective of preventive medicine, *ADH1B* may be important in screening and risk prediction in OPSCCs.

## AUTHOR CONTRIBUTIONS

**Huei‐Tzu Chien:** Conceptualization (lead); formal analysis (lead); methodology (lead); writing – original draft (lead). **Chia‐Lung Tsai:** Conceptualization (lead); software (supporting); writing – original draft (lead). **Chi‐Kuan Young:** Data curation (lead); investigation (lead); methodology (supporting); resources (lead); writing – review and editing (lead). **Yun‐Shien Lee:** Software (lead); validation (lead); visualization (lead); writing – review and editing (supporting). **Chun‐Ta Liao:** Data curation (lead); investigation (supporting); resources (lead); writing – review and editing (lead). **Chih‐Ching Yeh:** Investigation (supporting); software (supporting); writing – review and editing (supporting). **Angel Chao:** Investigation (supporting); methodology (lead); validation (supporting); writing – review and editing (supporting). **Kai‐Lun Cho:** Data curation (lead); investigation (lead); methodology (supporting). **Ching‐Han Chen:** Data curation (supporting); investigation (supporting); methodology (supporting). **Shiang‐Fu Huang:** Conceptualization (lead); data curation (lead); funding acquisition (lead); investigation (lead); methodology (lead); project administration (lead); resources (supporting); writing – original draft (supporting); writing – review and editing (lead).

## FUNDING INFORMATION

This study was supported by grants CMRPG3L0861, CMRPG3J0593, CMRPG3M1101, and CMRPG3M1102 from Chang Gung Memorial Hospital and grant MOST 110‐2314‐B‐182‐045‐MY3 from the Ministry of Science and Technology, Executive Yuan, Taiwan.

## CONFLICT OF INTEREST STATEMENT

The authors declare no conflicts of interest.

## ETHICS STATEMENT AND CONSENT

This study was performed in compliance with the Declaration of Helsinki. The study was approved by the Institutional Review Board of Chang Gung Memorial Hospital (IRB no. 201800439B0) and written consent was obtained from all participants. Ethnically‐ and geographically matched healthy controls from a biobank as a nationwide population study was approved by Taipei Medical University (TMU‐201805076).

## Supporting information


Figure S1.

Figure S2.

Table S1.

## Data Availability

Data were deposited in Gene Expression Omnibus (GEO): https://www.ncbi.nlm.nih.gov/geo/query/acc.cgi?acc=GSE218224. Reviewer toke: srilciawrlkvlmr. The data are open for sharing.
