# Supplementary figures and images for: Single‐nucleotide polymorphism at alcohol dehydrogenase 1B: A susceptible gene marker in oro‐/hypopharyngeal cancers from genome‐wide association study
Source: Cancer Med. 2023 Sep 14;12(18):19174–87. doi: 10.1002/cam4.6506 (PMC10557853; doi:10.1002/cam4.6506)

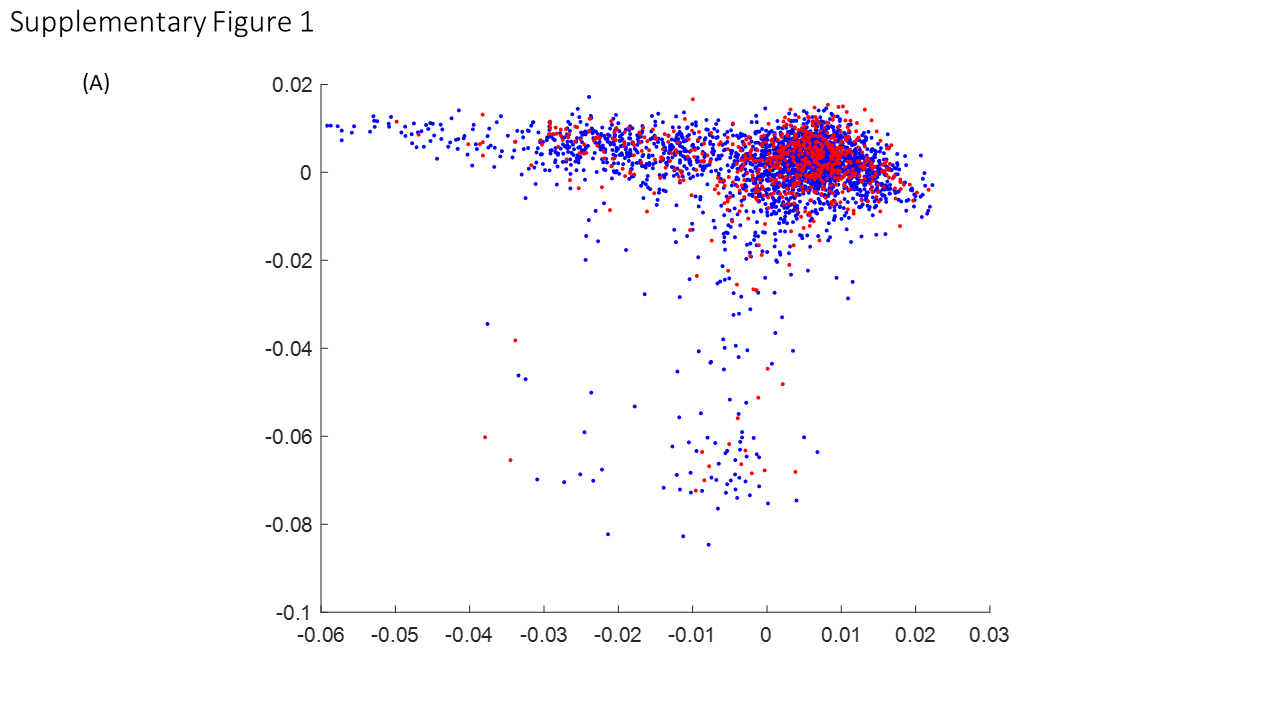

Supplement: Supplementary file 1 — Figure S1. [file CAM4-12-19174-s003.zip › Supple Fig 1A.TIF]

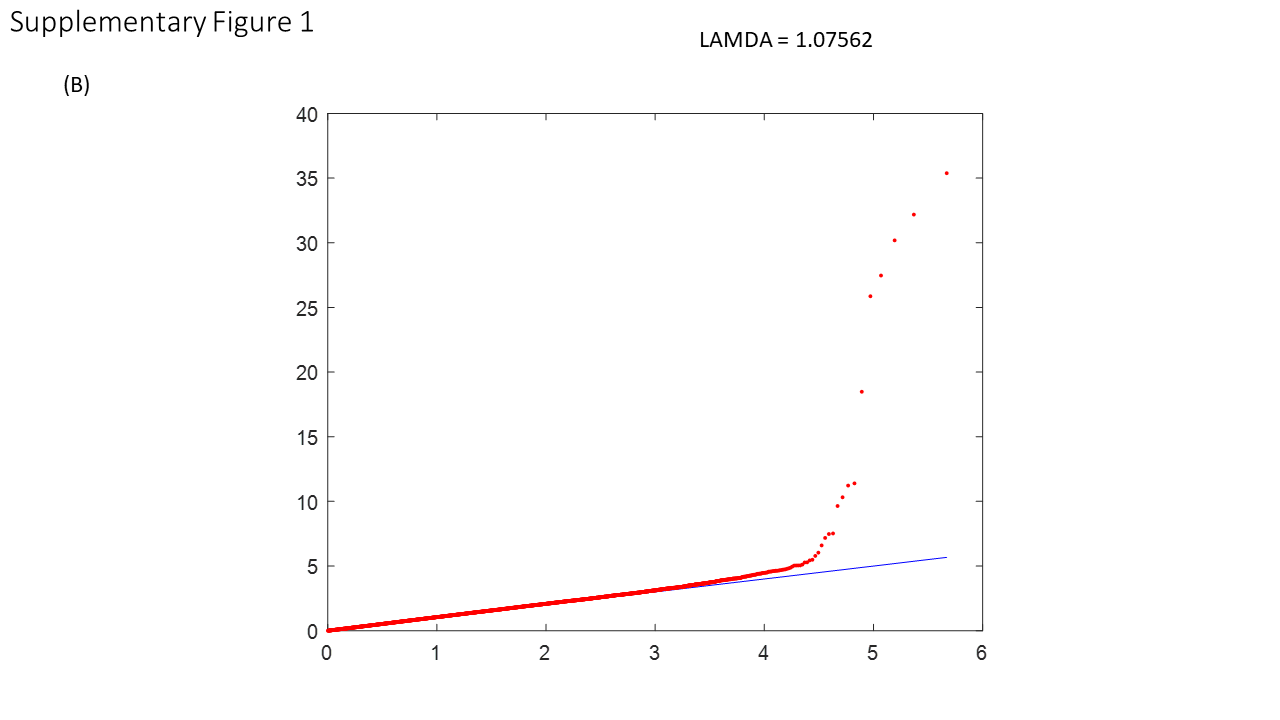

Supplement: Supplementary file 1 — Figure S1. [file CAM4-12-19174-s003.zip › Supple Fig 1B.TIF]

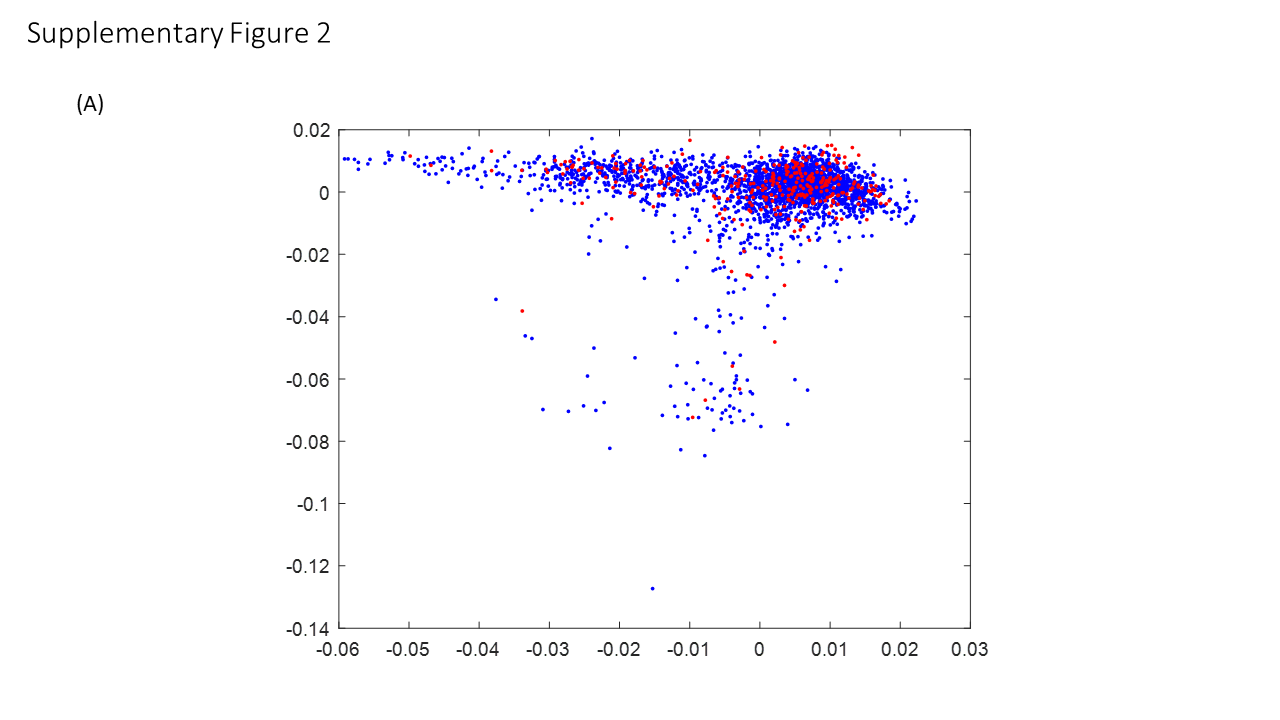

Supplement: Supplementary file 2 — Figure S2. [file CAM4-12-19174-s002.zip › Supple Fig 2A.TIF]

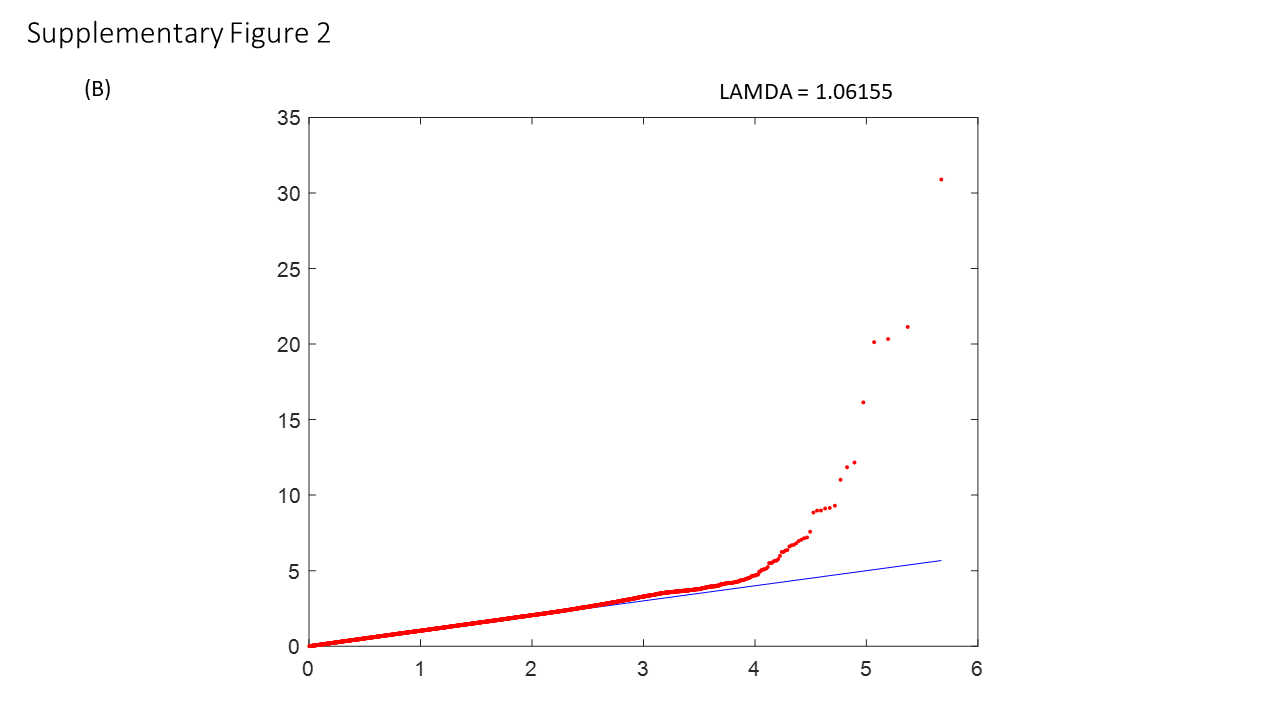

Supplement: Supplementary file 2 — Figure S2. [file CAM4-12-19174-s002.zip › Supple Fig 2B.TIF]
