# Supplementary material for: Single‐nucleotide polymorphism at alcohol dehydrogenase 1B: A susceptible gene marker in oro‐/hypopharyngeal cancers from genome‐wide association study
Source: Cancer Med. 2023 Sep 14;12(18):19174–87. doi: 10.1002/cam4.6506 (PMC10557853; doi:10.1002/cam4.6506)
Supplement: Supplementary file 3 — Table S1. [file CAM4-12-19174-s001.docx]

Supplementary Table 1.

| #CHROM | POS | ID | REF | ALT | A1 | OR | LOG(OR)_SE | L95 | U95 | P | Func.refGene | Gene.refGene |
| --- | --- | --- | --- | --- | --- | --- | --- | --- | --- | --- | --- | --- |
| 7 | rs74957563 | 77740777 | A | G | G | 6.93E-13 | 2.95311 | 2.19745 | 3.96862 | 6.93E-13 | intronic | RSBN1L |
| 4 | 99318162 | rs1229984 | T | C | C | 1.8277 | 0.085151 | 1.54676 | 2.15966 | 1.42E-12 | exonic | ADH1B |
| 9 | 40243677 | Affx-116457317 | C | G | G | 957.767 | 1.0078 | 132.867 | 6904.01 | 9.66E-12 | intronic | ANKRD20A2;  ANKRD20A3 |
| 4 | 99317841 | rs2075633 | C | T | T | 1.803 | 0.094768 | 1.49737 | 2.17101 | 4.97E-10 | intronic | ADH1B |
| 4 | 99280582 | rs975833 | C | G | G | 1.78839 | 0.094301 | 1.4866 | 2.15145 | 7.07E-10 | ncRNA_intronic | LOC100507053 |
| 4 | 99307309 | rs1042026 | C | T | T | 1.78494 | 0.094189 | 1.48405 | 2.14683 | 7.68E-10 | UTR3 | ADH1B |
| 4 | 99308253 | rs10005290 | C | A | A | 1.7785 | 0.094349 | 1.47824 | 2.13976 | 1.04E-09 | intronic | ADH1B |
| 4 | 99316676 | rs11730075 | G | A | A | 1.78328 | 0.094823 | 1.48083 | 2.1475 | 1.06E-09 | intronic | ADH1B |
| 4 | 99317256 | rs2066701 | A | G | G | 1.76916 | 0.094259 | 1.47073 | 2.12815 | 1.43E-09 | intronic | ADH1B |
| 19 | 13809063 | Affx-15570097 | G | T | T | 2.32607 | 0.151743 | 1.72767 | 3.13175 | 2.65E-08 | intronic | ZSWIM4 |
| 4 | 99221145 | Affx-23009917 | A | C | C | 1.64864 | 0.092427 | 1.37547 | 1.97605 | 6.33E-08 | ncRNA_intronic | LOC100507053 |
| 4 | 99310445 | rs28914783 | C | T | T | 1.86647 | 0.115816 | 1.48743 | 2.34209 | 7.11E-08 | intronic | ADH1B |
| 4 | 99274658 | rs12512110 | G | T | T | 1.95236 | 0.125144 | 1.52769 | 2.49507 | 8.99E-08 | ncRNA_intronic | LOC100507053 |
